# Supplementary material for: Reconstructing Earth’s atmospheric oxygenation history using machine learning
Source: Nat Commun. 2022 Oct 4;13:5862. doi: 10.1038/s41467-022-33388-5 (PMC9532422; doi:10.1038/s41467-022-33388-5)
Supplement: Supplementary file 1 — Supplementary Information [file 41467_2022_33388_MOESM1_ESM.pdf]

Supplementary information for:

Reconstructing Earth's Atmospheric Oxygenation History Using Machine Learning

Guoxiong Chen[1], Qiuming Cheng[1,2,3*], Timothy W. Lyons[4], Jun Shen[1], Frits Agterberg[5], Ning Huang[1], Molei Zhao[2]

[1] State Key Laboratory of Geological Processes and Mineral Resources, China University of Geosciences, Wuhan, 430074, China.

[2] State Key Laboratory of Geological Processes and Mineral Resources, China University of Geosciences, Beijing, 10083, China.

[3] School of Earth Science and Engineering, Sun Yat-sen University, Zhuhai, 51900, China.

[4] Department of Earth and Planetary Sciences, University of California, Riverside, California, 92521, USA.

[5] Geological Survey of Canada, 601 Booth Street, Ottawa, Ontario, K1A 0E8, Canada.

Corresponding author: Qiuming Cheng (qiuming.cheng@iugs.org)

This supplementary document contains the following information:

Supplementary Figure 1. Histogram of mafic igneous geochemical concentration.

Supplementary Figure 2. Filtering outliers in mafic igneous geochemistry data.

Supplementary Figure 3. Time series record of global mean mafic geochemistry composition.

Supplementary Figure 4. Cluster analysis of mafic geochemistry composition data.

Supplementary Figure 5. SOM analysis of mafic igneous geochemistry data.

Supplementary Figure 6. Principal component analysis of mafic igneous geochemistry data.

Supplementary Figure 7. Parameterization and optimization in SVR modeling.

Supplementary Figure 8. SVR modeling test with different uncertainties in training labels.

Supplementary Figure 9. SVR modeling test with constraints in boring billion and GOE.

Supplementary Figure 10. SVR modeling test using different training datasets.

Supplementary Figure 11. PCA and SVR modeling of CLR-transformed composition data.

Supplementary Figure 12. O_2 modeling curves using different machine learning methods.

Supplementary Figure 13. Feature importance of all elements in RF modeling.

Supplementary Figure 14. Sedimentary-hosted paleo-oxybarometers through time.

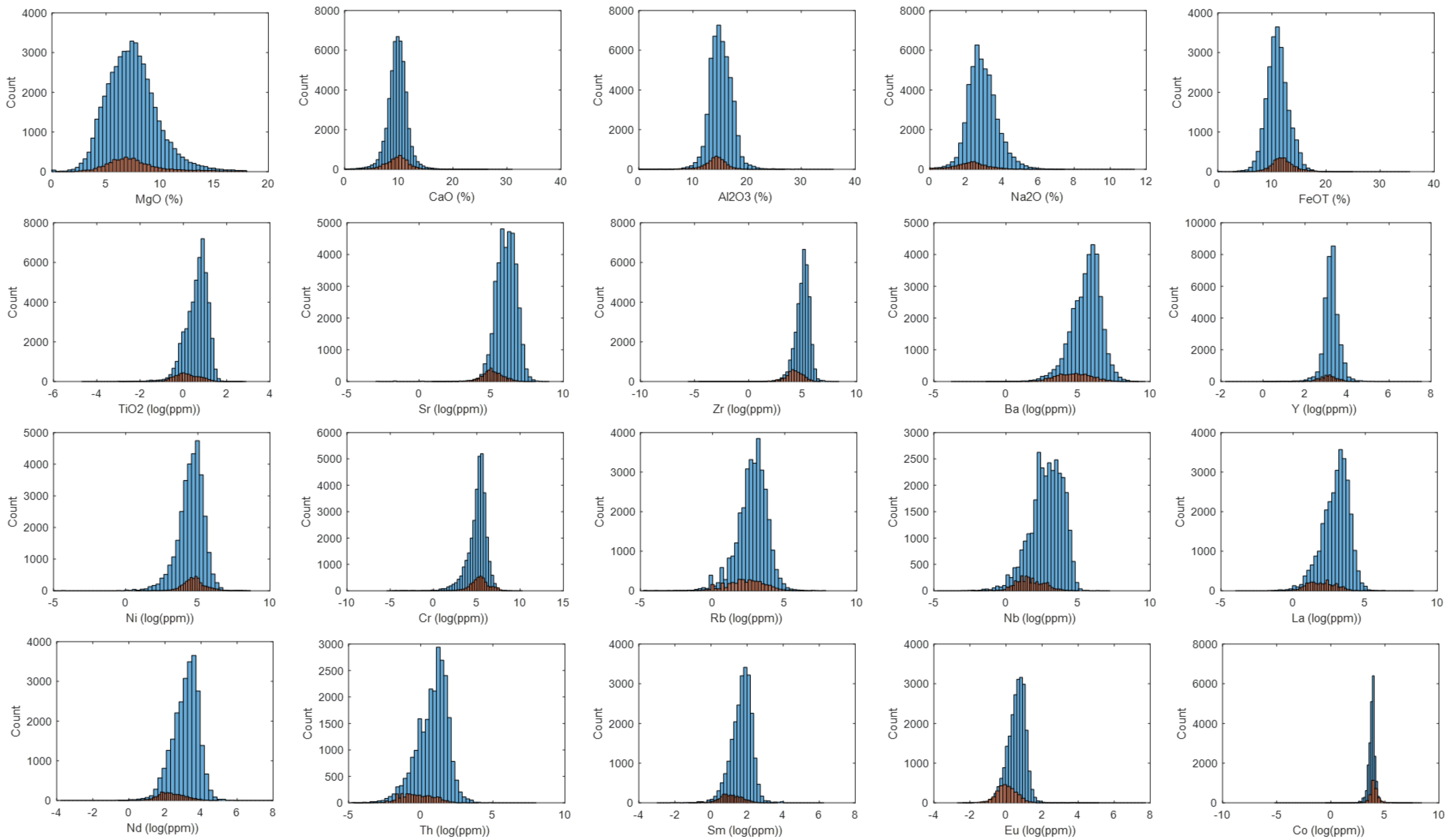

Supplementary Figure 1

Histogram of mafic igneous geochemical concentration. Frequency distribution of mafic igneous geochemical concentration (few examples) during the Phanerozoic (blue) and Precambrian (brown) time. Trace elements often follow lognormal distribution, while major elements follow normal distribution.

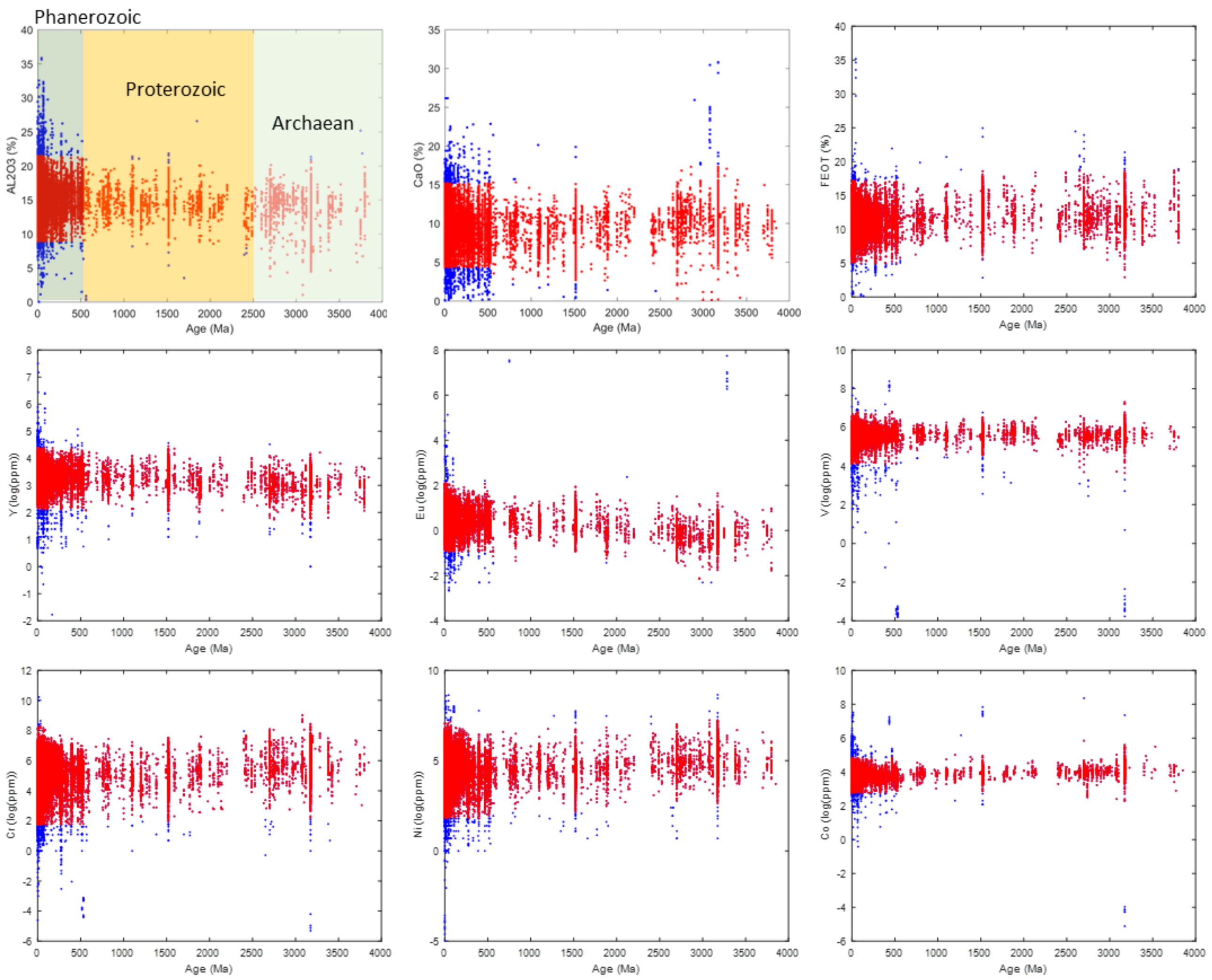

Supplementary Figure 2

Filtering outliers in mafic igneous geochemistry data by using mean ± 3δ thresholding. Logarithm transformation was applied to trace elements data for outliers filtering. The bule points indicate the filtered samples while red points are for calculation.

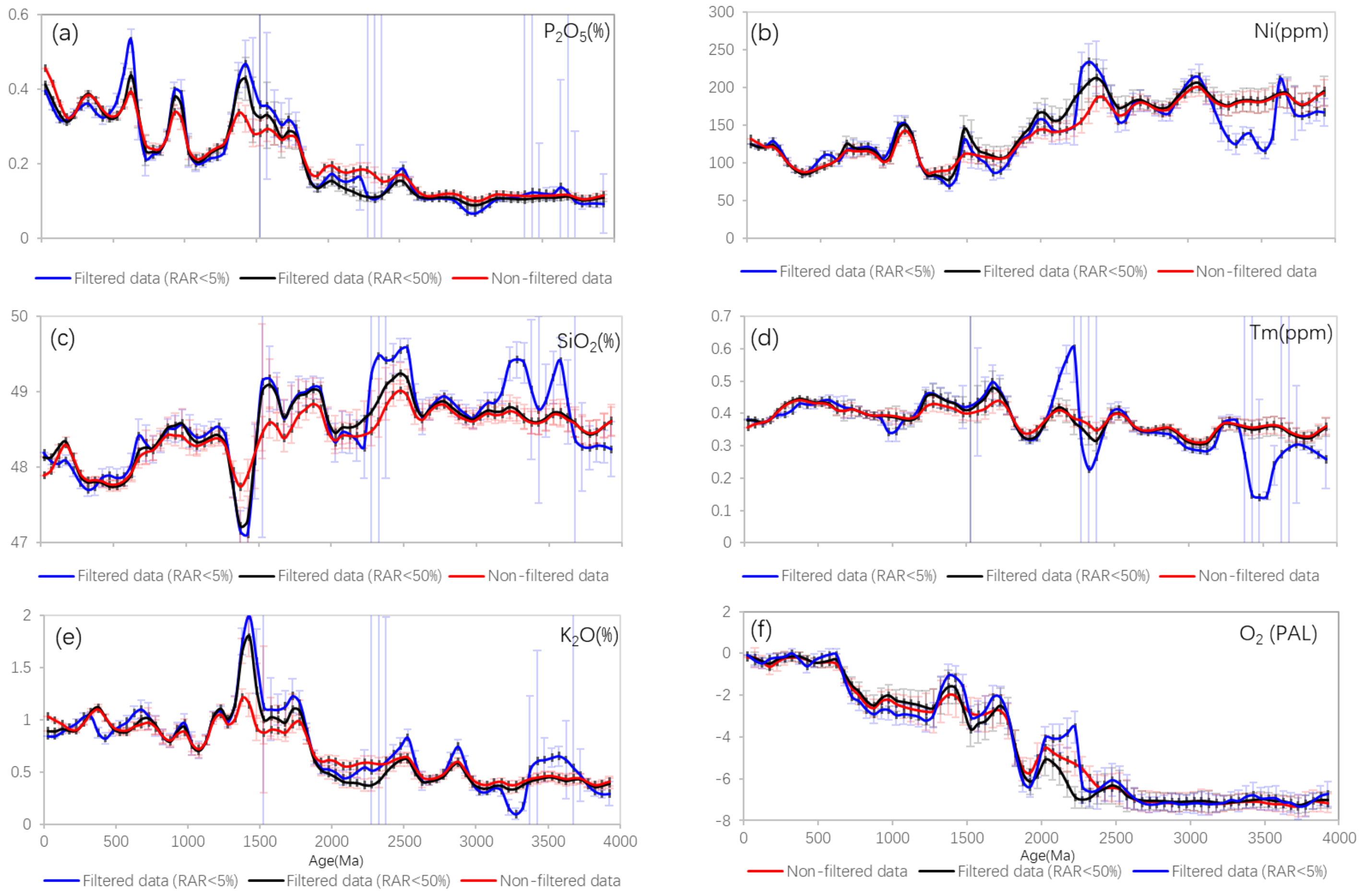

Supplementary Figure 3

Time series record of global mean mafic geochemistry composition. (a-e) Time series records of mean geochemical concentration obtained by the weighted bootstrap sampling method[1] from filtered data with RAR[2] <5% (blue line) and RAR<50% (black line), and non-RAR filtered dataset (red line). (f) Atmospheric O_2 modeling patterns obtained from filtered and non-RAR filtered datasets. The error bar shows 2 standard deviation (2-SD) uncertainties.

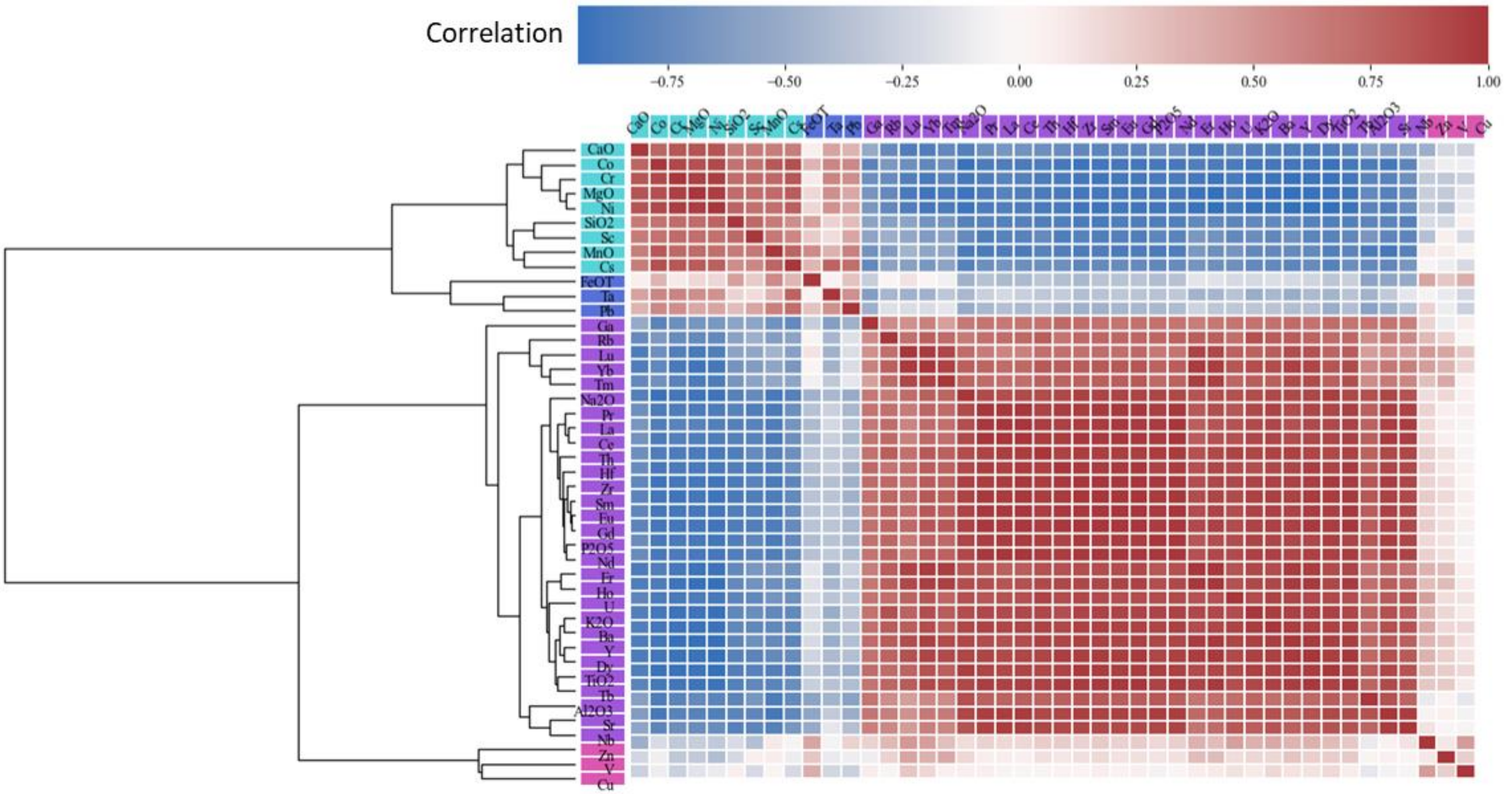

Supplementary Figure 4

Cluster analysis of mafic geochemistry composition data. Correlation matrix and cluster analysis of mafic composition spanning 44 elements from mafic igneous geochemical composition data; each square shows the Pearson's correlation coefficient for a pair of elements, and the intensity of the red or blue color indicates the value of correlation, as shown on the color bar; hierarchical clusters are represented by a cluster tree on the left panel.

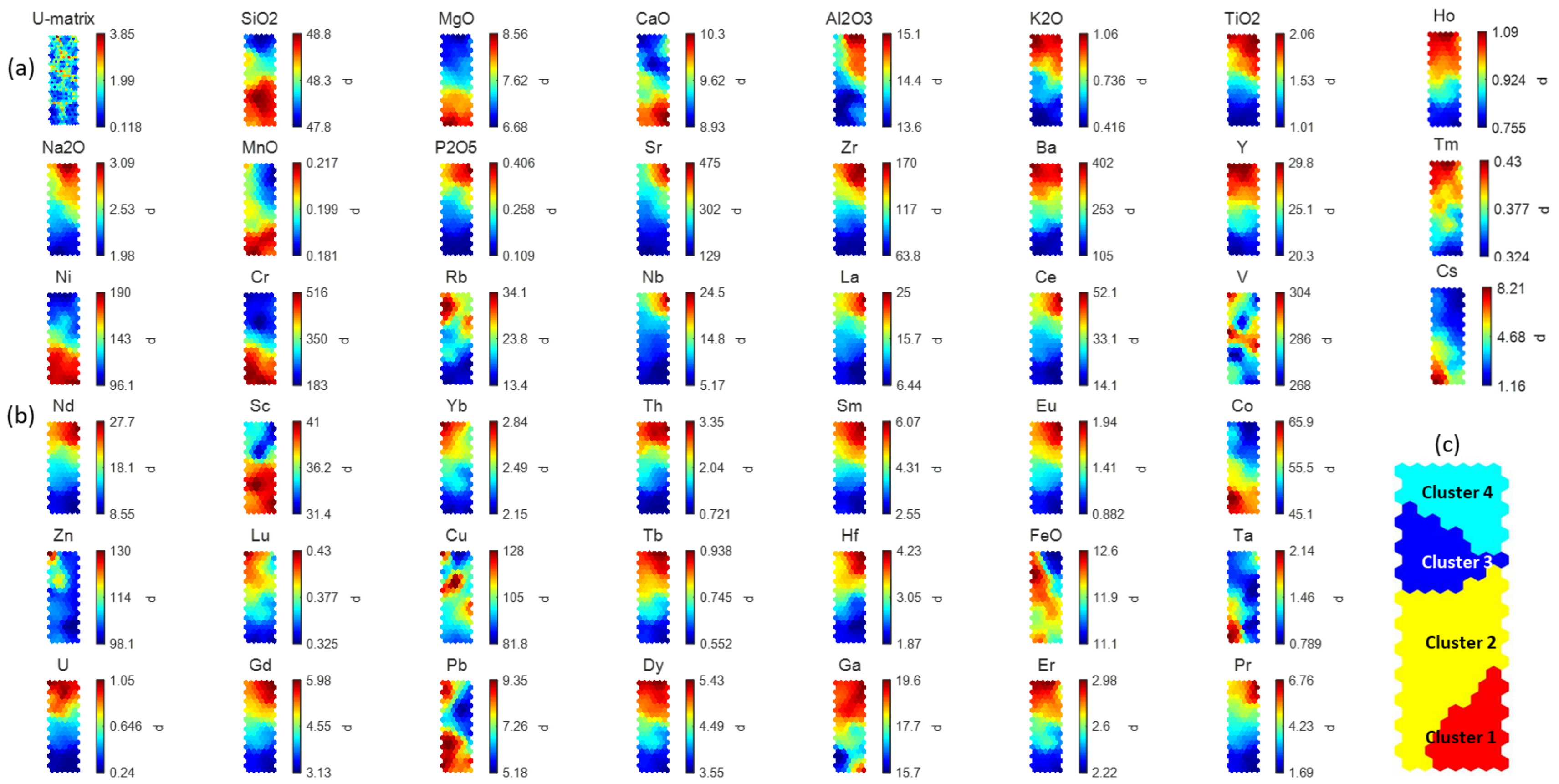

Supplementary Figure 5

SOM analysis of mafic igneous geochemistry data. (a) U-matrix of the trained SOM, uniform areas of low value indicate clusters while high values indicate cluster border, and color bar is the distance between the prototype vectors. (b) Component plots of 44 elements in SOM analysis. Component plots show the principal relations visually identified between the 44 variables. Warm colors represent high contribution to the SOM pattern, while cool colors represent low contribution. (c) SOM classification result of global mafic igneous geochemistry time series data, with colors coded in light blue, dark blue, yellow, and red for clusters 1, 2, 3 and 4, respectively.

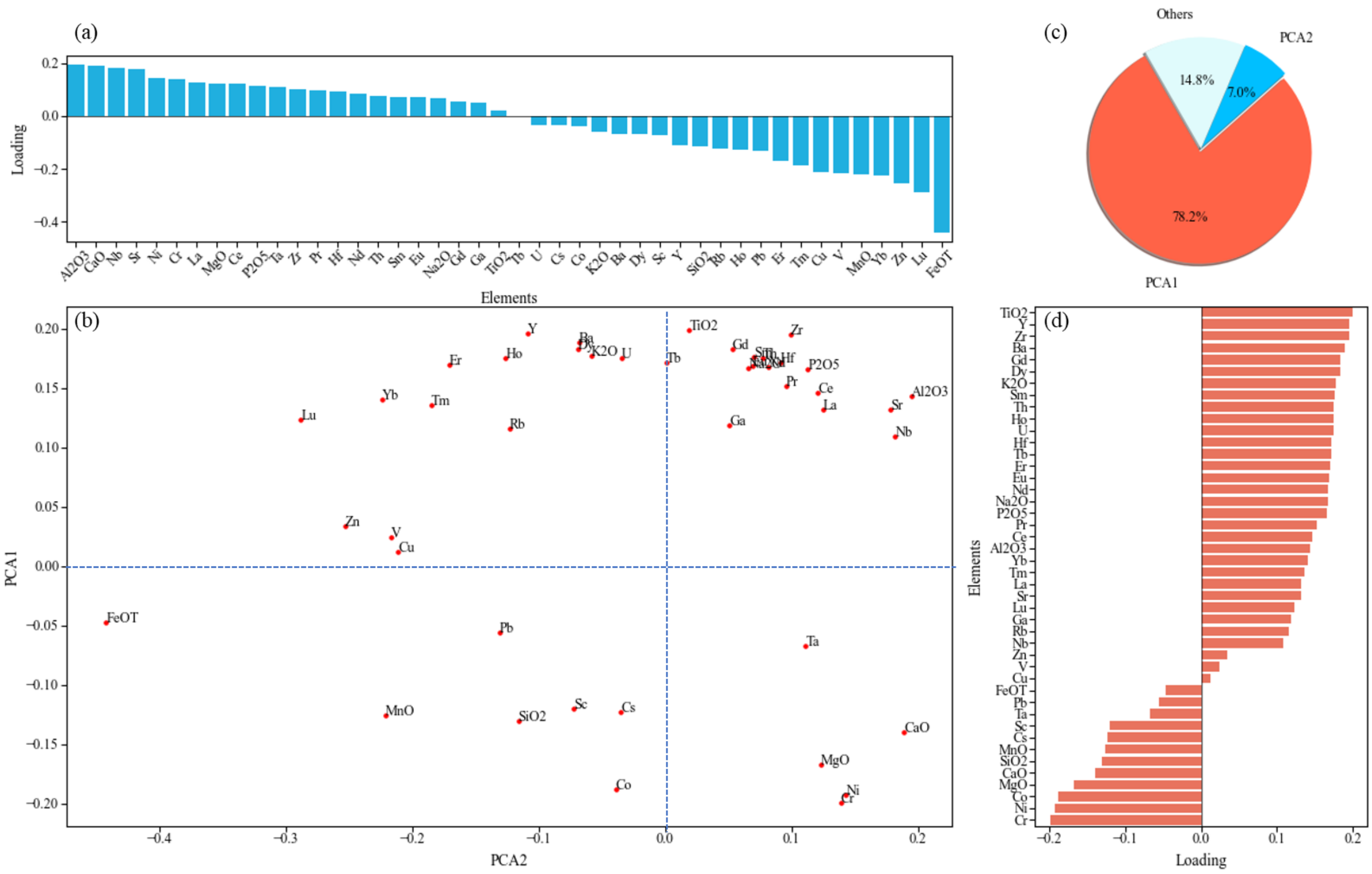

Supplementary Figure 6

PCA analysis of mafic igneous geochemical data. (a) and (d) show the element loadings of PCA2 and PCA1, respectively. (b) Variance contribution of PCA1, PCA2 and rest principal components. (c) PCA1 loadings vs. PCA2 loadings for all elements.

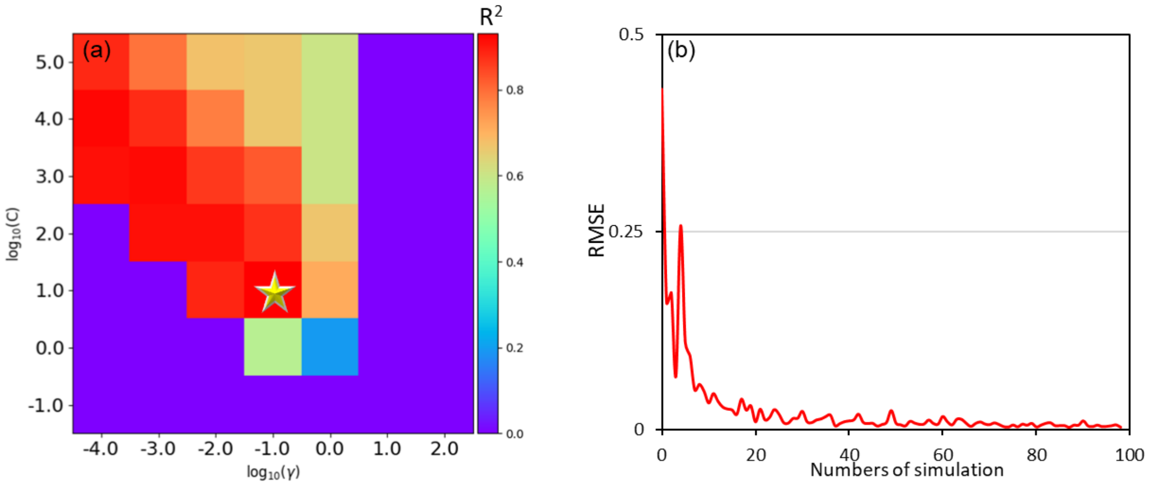

Supplementary Figure 7

Parameterization and optimization in Support Vector Regression (SVR) modeling. (a) Illustration shows one example of prediction accuracy in variation with RBF function parameter γ and coefficient of penalty C for SVR, and the optimal parameters (γ = 0.1, C = 10) used in the calculation are determined by the maximum of $R^2 = 0.92$. (b) The root-mean-square-error (RMSE) curve of SVR modeling with Monte Carlo simulation.

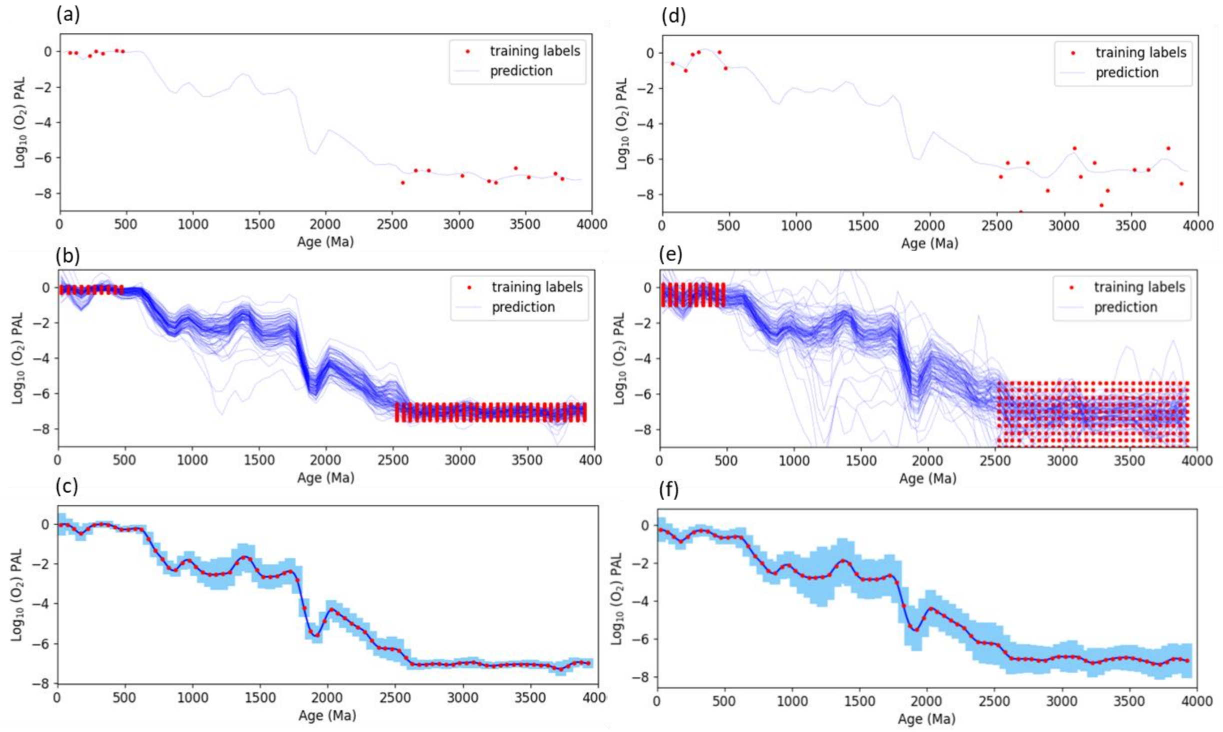

Supplementary Figure 8

SVR modeling test with different uncertainties in training labels. Left panels (a) to (c) show the prediction of atmospheric O_2 variation through Monte Carlo simulation, using random labels (including numbers and values) ranging from 0.5 to 1.2 PAL post 0.5 Ga and $10^{-6.5}$ to $10^{-7.5}$ PAL pre 2.5 Ga, while right panels (d) to (f) show the prediction using random labels ranging from 0.1 to 2 PAL post 0.5 Ga, and 10^{-5} to 10^{-9} PAL pre 2.5 Ga. Illustrations (a) and (d) show one example of SVR predictions, while (b) and (e) show 100 curves of SVR predictions. (c) and (f) show the time series record of mean O_2 prediction through SVR and Monte Carlo simulation. The error bar shows 2 standard deviation (2-SD) uncertainties.

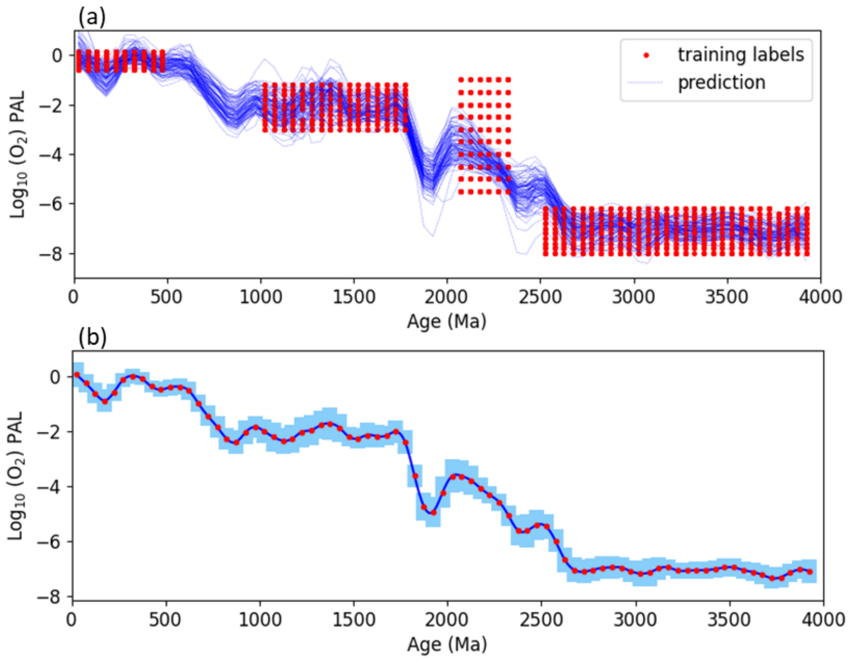

Supplementary Figure 9

SVR modeling test with constraints in boring billion and GOE. (a) All SVR modeling curves of atmospheric O_2 content using global mafic igneous geochemistry data through Monte Carlo simulations (*n* = 100) with additional informed labels during the "boring" billion and the "oxygen overshoot" of GOE. (b) Estimated atmospheric O_2 variation through time using mean value of 1000 simulations, the error bar shows 2 standard deviation (2-SD) uncertainties

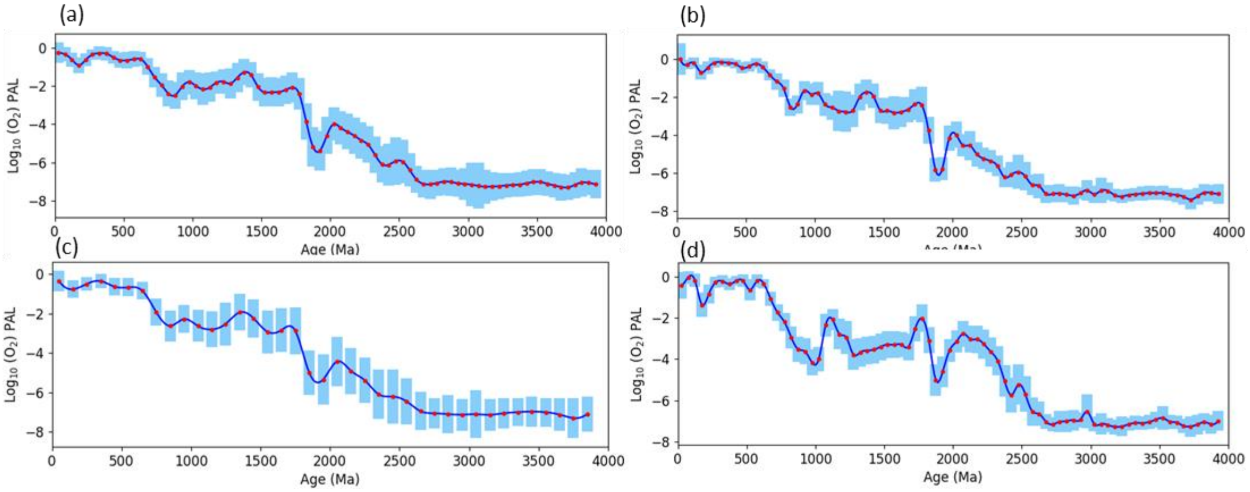

Supplementary Figure 10

SVR modeling test using different training datasets. (a) SVR modeling only using the highly correlated elements as suggested by cluster analysis in supplementary Fig. 4 (i.e., precluding FeO_T, Ta, Pb, Cu, V and Zn). (b) SVR modeling of atmospheric O_2 contents using mafic igneous geochemical time series data prepared with 50 Myr bin and uncertainty age = 50 Ma. (c) SVR modeling of atmospheric O_2 contents using mafic igneous geochemical time series data prepared with 100 Myr bin and uncertainty age = 100 Ma. (d) SVR modeling of atmospheric O_2 contents using the time series dataset of Keller and Schoene[1]. The error bar shows 2 standard deviation (2-SD) uncertainties.

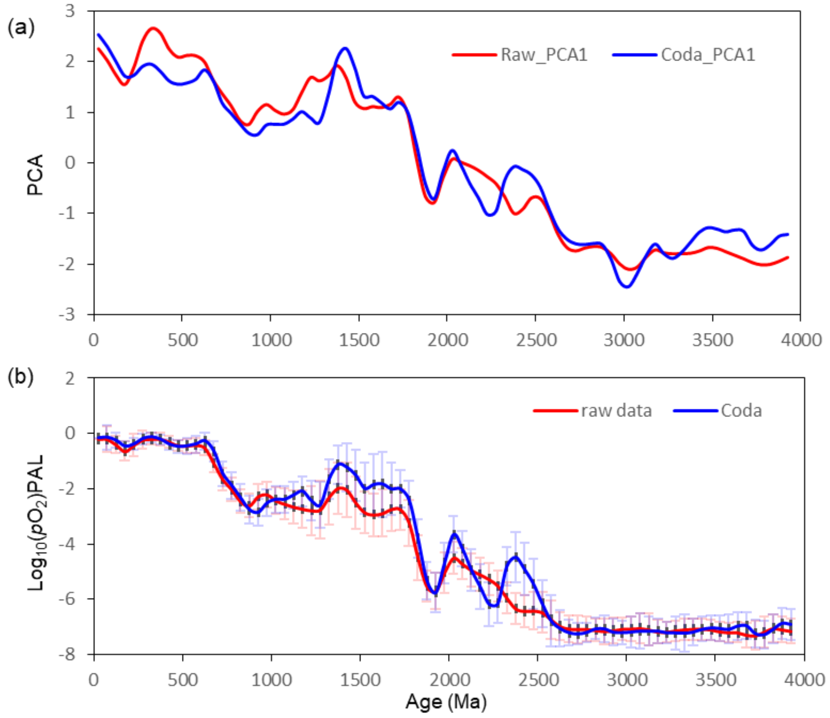

Supplementary Figure 11

PCA and SVR modeling of Centered Log-Ratio (CLR)[3] transformed global mafic composition data. (a) The first principal component in PCA of global mafic composition data. (b) Atmospheric O_2 prediction using SVR method with CLR transformed mafic composition data. The error bar shows 2 standard deviation (2-SD) uncertainties.

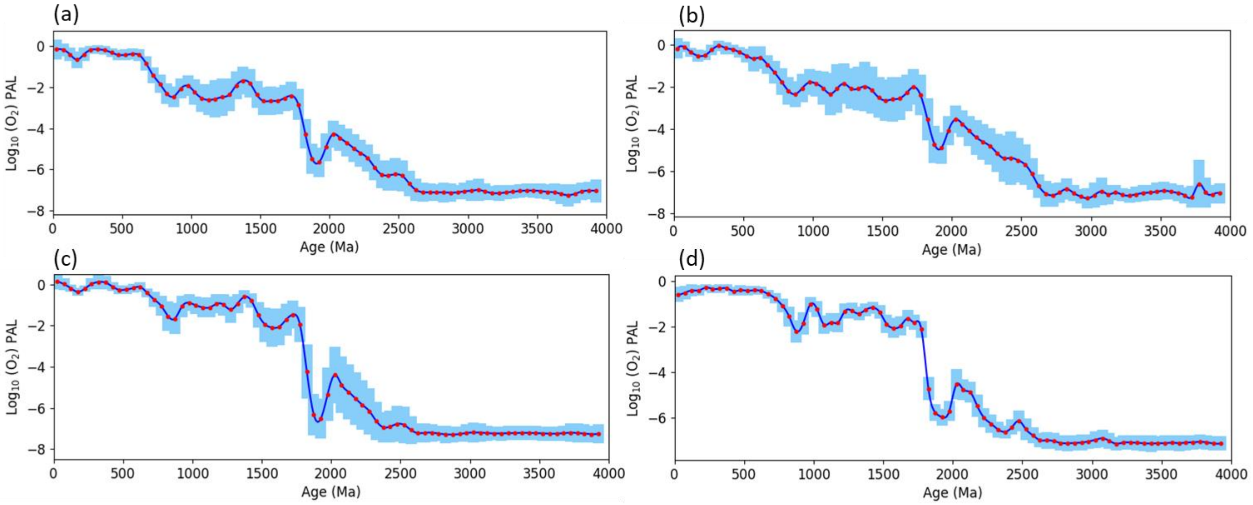

Supplementary Figure 12

O_2 modeling curves using different machine learning methods. SVR modeling of atmospheric oxygen level using (a) min-max scaling and (b) log scaling normalization methods. Also shown are reconstruction of atmospheric oxygen content using (c) artificial neural network and (d) random forests algorithms, respectively. The error bar shows 2 standard deviation (2-SD) uncertainties.

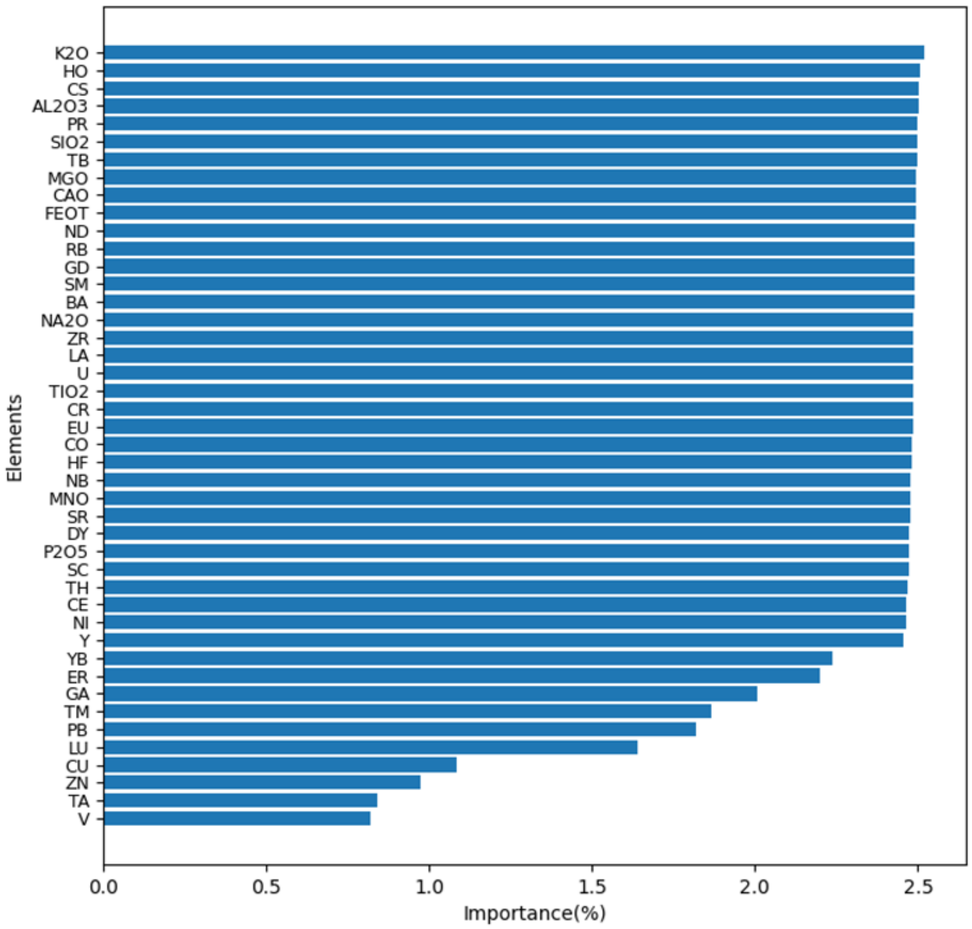

Supplementary Figure 13

Feature importance of all elements in Random Forests modeling. Averaging feature importance of all elements after 10000 stochastic simulations, illustrating how each composition variable influences the final prediction model of atmospheric oxygen levels.

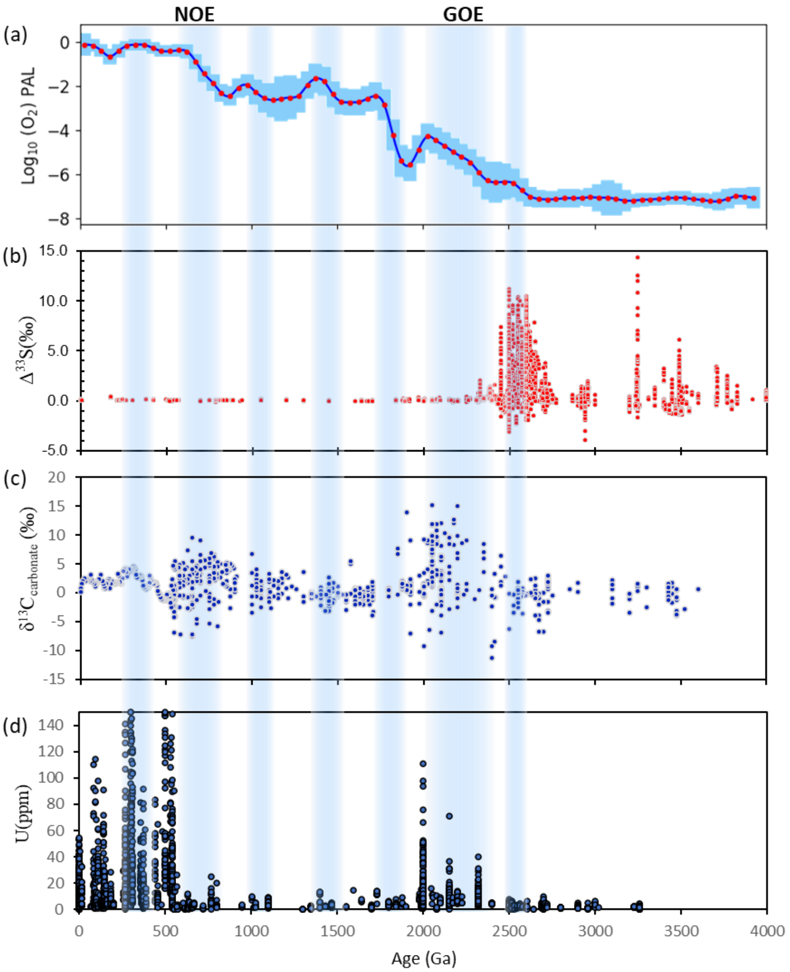

Supplementary Figure 14

Sedimentary-hosted paleo-oxybarometers through time. (a) The emerge curve of atmospheric O_2 level in the present study, the error bar shows 2 standard deviation (2-SD) uncertainties. (b) Pyrite and sulfate $\Delta^{33}S$ data[4]. (c) Carbonate $\delta^{13}C$ data[5]. (d) Uranium concentrations from marine shales[6].

Supplementary references

1. Keller, C. B. & Schoene, B. Statistical geochemistry reveals disruption in secular lithospheric evolution about 2.5 Gyr ago. *Nature* **485**, 490-493 (2012).
2. Doucet, L. S., Gamaleldien, H. & Li, Z.-X. Pitfalls in using the geochronological information from the EarthChem Portal for Precambrian time-series analysis. *Precambrian Research* **369**, 106514 (2022).
3. Greenacre, M. Compositional data analysis. *Annual Review of Statistics and its Application* **8**, 271-299 (2021).
4. Killingsworth, B. A. *et al.* Constraining the rise of oxygen with oxygen isotopes. *Nature communications* **10**, 1-10 (2019).
5. Krissansen-Totton, J., Buick, R. & Catling, D. C. A statistical analysis of the carbon isotope record from the Archean to Phanerozoic and implications for the rise of oxygen. *American Journal of Science* **315**, 275-316 (2015).
6. Partin, C. A. *et al.* Large-scale fluctuations in Precambrian atmospheric and oceanic oxygen levels from the record of U in shales. *Earth and Planetary Science Letters* **369**, 284-293 (2013).
